# Percutaneous Intramedullary Nailing of Complex Humeral Shaft Fractures: A Retrospective Case Series

**DOI:** 10.7759/cureus.32999

**Published:** 2022-12-27

**Authors:** Parke W Hudson, Matthew T Gulbrandsen, Joseph N Liu, Brent A Ponce, Wesley P Phipatanakul

**Affiliations:** 1 Orthopaedic Surgery, Loma Linda University Medical Center, Loma Linda, USA; 2 Orthopaedic Surgery, University of Southern California, Los Angeles, USA; 3 Orthopaedic Surgery, University of Alabama at Birming, Birmingham, USA

**Keywords:** proximal humerus fracture, percutaneous fixation, orthopaedic trauma, intramedullary nail, humerus fracture

## Abstract

Background: Humeral diaphyseal fractures have been traditionally stabilized with plates and screws. However, surgical morbidity can be quite extensive, particularly in more complex segmental and comminuted fracture patterns. An intramedullary nail (IMN) has the biomechanical advantage of being a load-sharing device and can be placed with a more minimally invasive technique. The purpose of this study was to evaluate the clinical and radiographic outcomes of complex humeral shaft fractures treated with an IMN utilizing a percutaneous surgical technique.

Methods: A retrospective review was performed on a consecutive series of patients who underwent treatment of a complex humeral shaft fracture with an IMN placed with a percutaneous technique. Clinical outcome scores and radiographic analysis were performed at a minimum one-year follow-up.

Results: Of the 14 patients included, 12 had clinical and radiographic follow-ups at one year. The majority (64%) were obese and involved polytrauma (50%), and 79% were AO Foundation/Orthopaedic Trauma Association (AO/OTA) type C fractures. Union after the index procedure was 93%, with one nonunion requiring a secondary operation. The average operative time was 103 minutes. There were no other complications or additional procedures. The mean clinical outcome scores included American Shoulder and Elbow Society (ASES): 78.2, Constant Score: 72.1, Single Assessment Numerical Evaluation (SANE): 81.9, and Penn Shoulder Score: 82.7.

Conclusion: This study demonstrates complex comminuted and segmental humeral shaft fractures in a higher-risk patient population can be effectively managed with IMN. Percutaneous placement of an IMN should be considered as a treatment option in complex humeral shaft fractures, particularly in patients with secondary comorbidities such as obesity and polytrauma.

## Introduction

Humeral diaphyseal fractures are relatively common in orthopaedic trauma, representing roughly 3% of all fractures, and can often be treated without surgical intervention [1]. Indications for surgical stabilization include polytrauma, open fractures, floating elbows, neurovascular injuries, and fractures in which adequate reduction cannot be maintained via closed management [2-4]. In cases of more complex fractures, such as comminuted or segmental patterns, plate fixation may need to span nearly the entire length of the humerus in order to adequately stabilize the fracture, which often results in significant surgical morbidity due to soft tissue dissection. In such circumstances, the utility of an intramedullary nail (IMN) is its ability to be a load-sharing device with a minimally invasive technique, resulting in less soft tissue disruption, less blood loss [2,5], lower incidence of radial nerve palsy [6-8], and decreased operative time [9].

Current literature descriptions of humeral IMN techniques include detachment or splitting of the deltoid and disruption of the rotator cuff [10,11]. This dissection can at times be cumbersome in obese patients, leading to unanticipated blood loss, which is especially concerning in polytrauma patients. Thus, the purpose of this study was to evaluate the clinical and radiographic outcomes of complex (multifragmentary, segmental, and long wedge) humeral shaft fractures treated with an IMN utilizing a percutaneous surgical technique. Our hypothesis is that this surgical approach will achieve adequate clinical and radiographic outcomes in patients with complex humeral shaft fractures.

## Materials and methods

**Figure 1:**
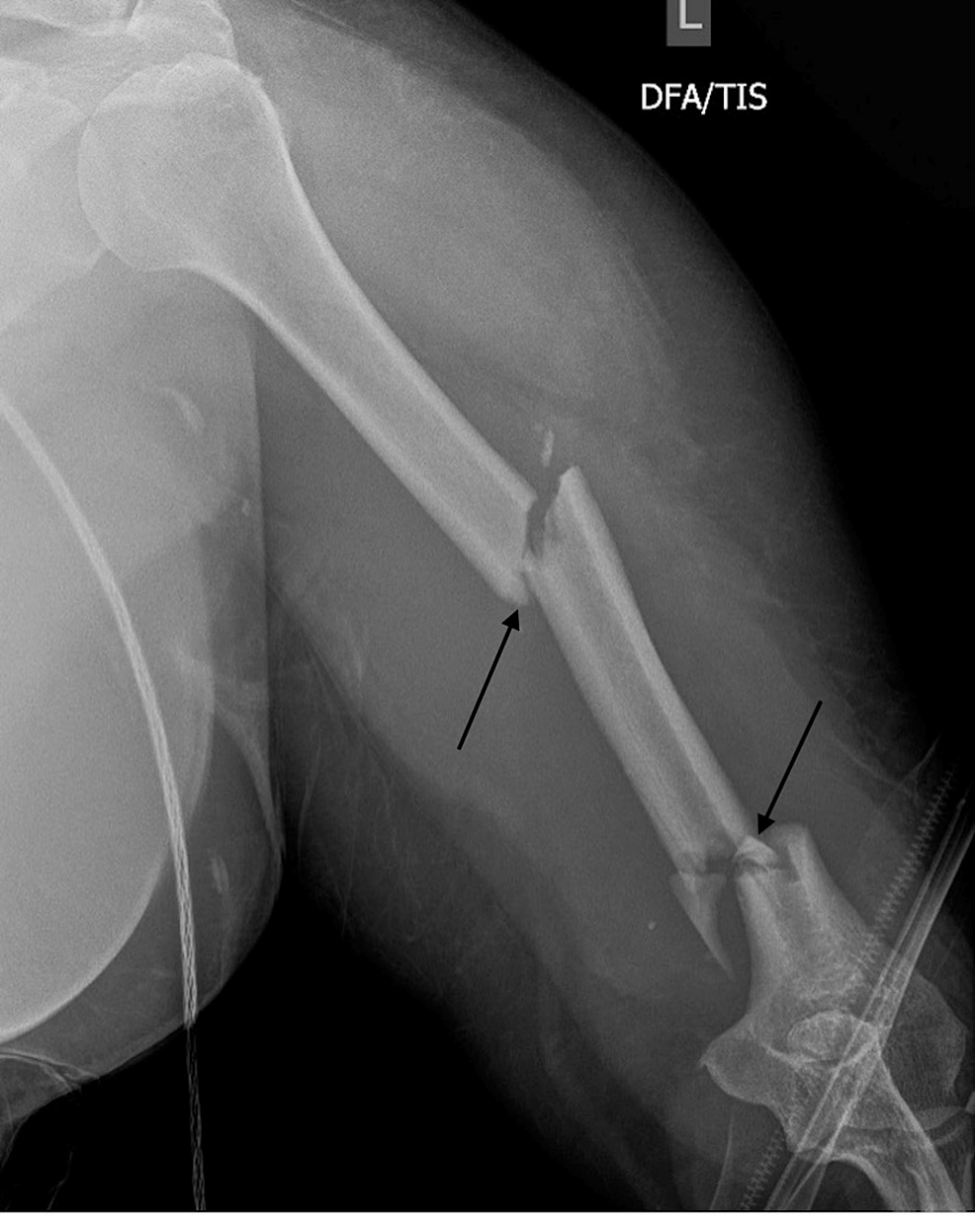
Anterior/posterior radiographs of a humerus demonstrate an example of a segmental fracture pattern with arrows to show the separate fractures.

**Figure 2:**
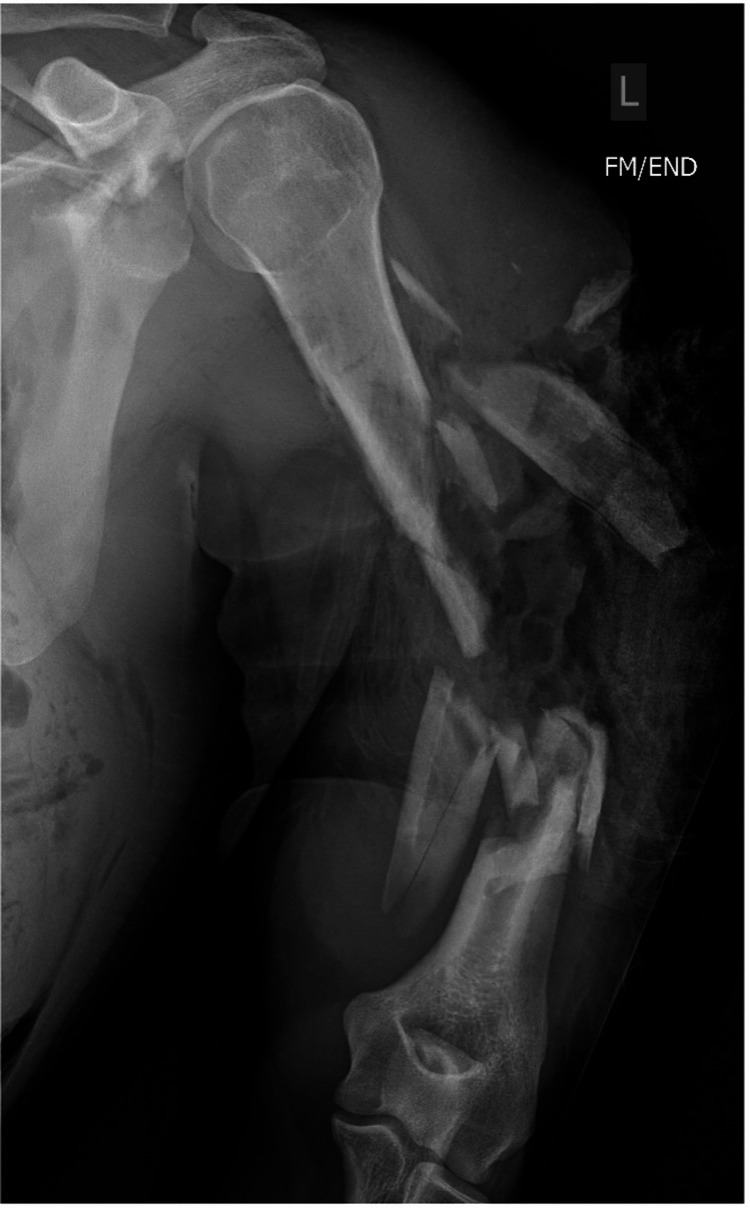
Anterior/posterior radiographs of a humerus demonstrate an example of a comminuted fracture pattern.

An IRB-approved (#5150199) retrospective chart and radiographic review were performed on 14 consecutive patients with humeral shaft fractures treated with a percutaneous IMN technique. Complex segmental (Figure 1) and comminuted (Figure 2) fractures were included. The primary indications for this procedure were patients with comminuted, long wedge, and segmental fracture patterns of AO Foundation/Orthopaedic Trauma Association (AO/OTA) types B and C [12]. Each patient’s age, body mass index, AO/OTA fracture type, American Society of Anesthesiologists classification, and concomitant fractures were recorded preoperatively. Polytrauma was defined as a concurrent long bone or pelvic fracture. Postoperatively, operative time (defined as the time after intubation until final dressing application), union rate, radial nerve palsies, follow-up time, and outcome scores were recorded. Clinical outcome measures included the American Shoulder and Elbow Society (ASES), Constant, Single Assessment Numeric Evaluation (SANE), and Penn Shoulder (PENN) scores. Descriptive statistics were performed on the collected data. All patients were followed to the radiographic union. Radiographic union was defined as bridging the callus on three of the four cortices of all major fracture fragments.

Surgical technique

The patient was placed supine on a flat Jackson radiolucent table with the C-arm brought in from the contralateral side. There are several potential advantages of supine positioning over beach chair or lateral positioning: (1) the C-arm technician can more easily maneuver around a completely flat radiolucent table; (2) not all facilities have a beach chair positioner table; (3) supine positioning is generally quicker and facilitates multi-site surgical care of polytrauma patients; (4) it can be used safely in patients with cervical spine precautions; and (5) there is less obstruction for proximal guide pin placement, particularly compared to beach chair positioning. The nonoperative contralateral upper extremity was securely tucked next to the body. It is important to ensure there are no arm boards above the umbilicus to allow adequate space for maneuvering the C-arm. For larger patients, arm boards were placed starting at the lower abdominal area and oriented distally if needed to support the patient’s body habitus. The operative extremity was preferably attached to a pneumatic arm holding device or alternatively placed on a sterilely covered, padded Mayo stand.

**Figure 3:**
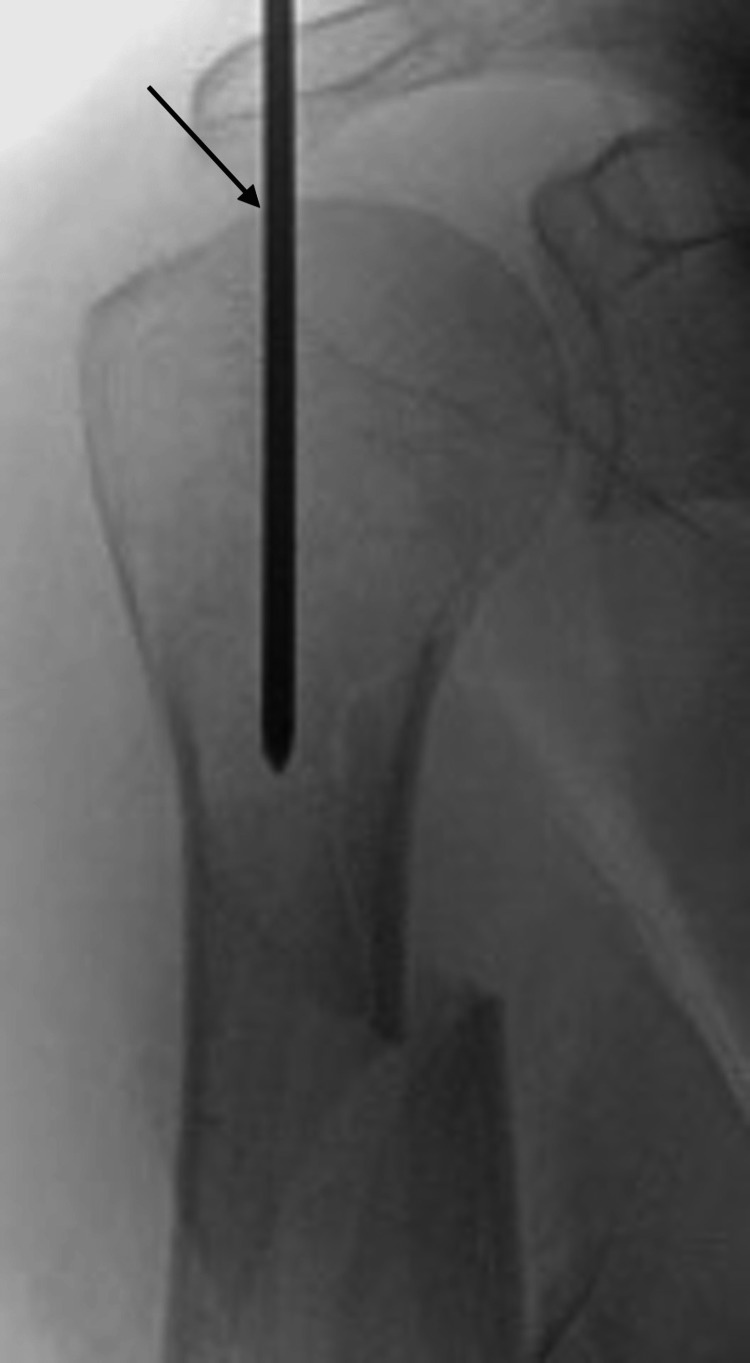
Fluoroscopic anterior/posterior image of an appropriate entry point (shown by the arrow) into the humeral head on the coronal plane. It is important to start medial to the greater tuberosity to avoid the tendinous portion of the rotator cuff.

**Figure 4:**
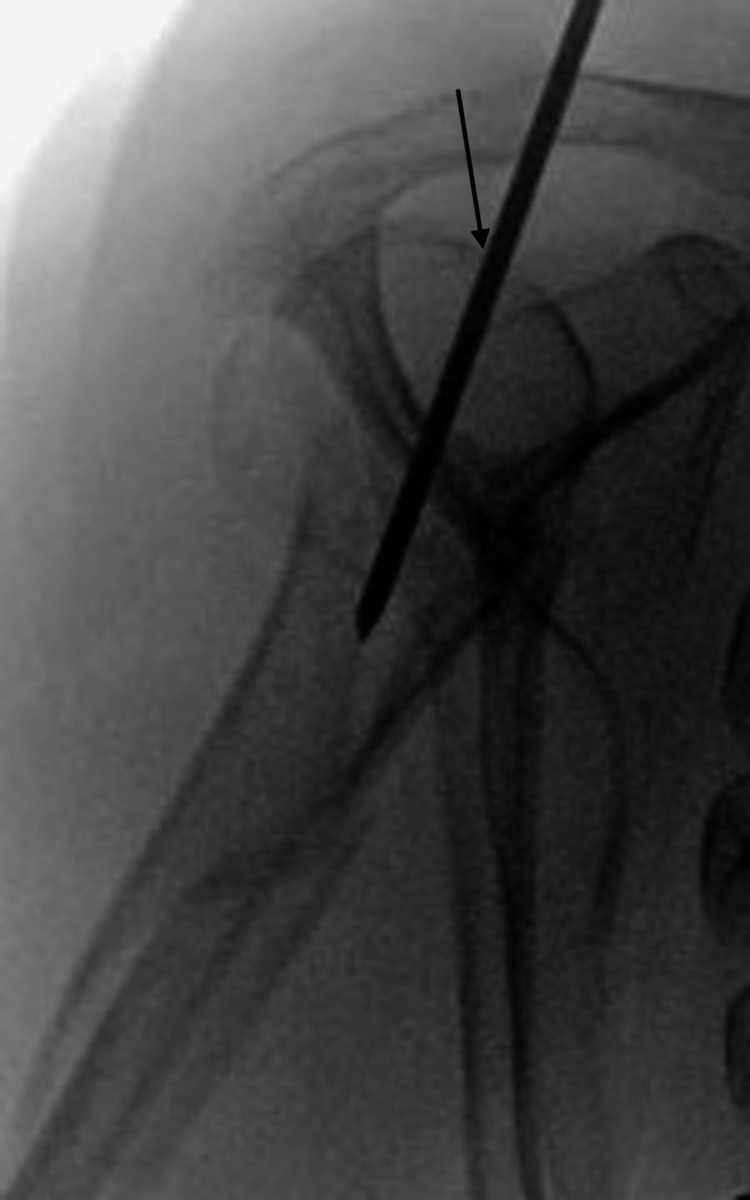
Fluoroscopic images demonstrating an appropriate entry point (shown by the arrow) in the sagittal plane.

**Figure 5:**
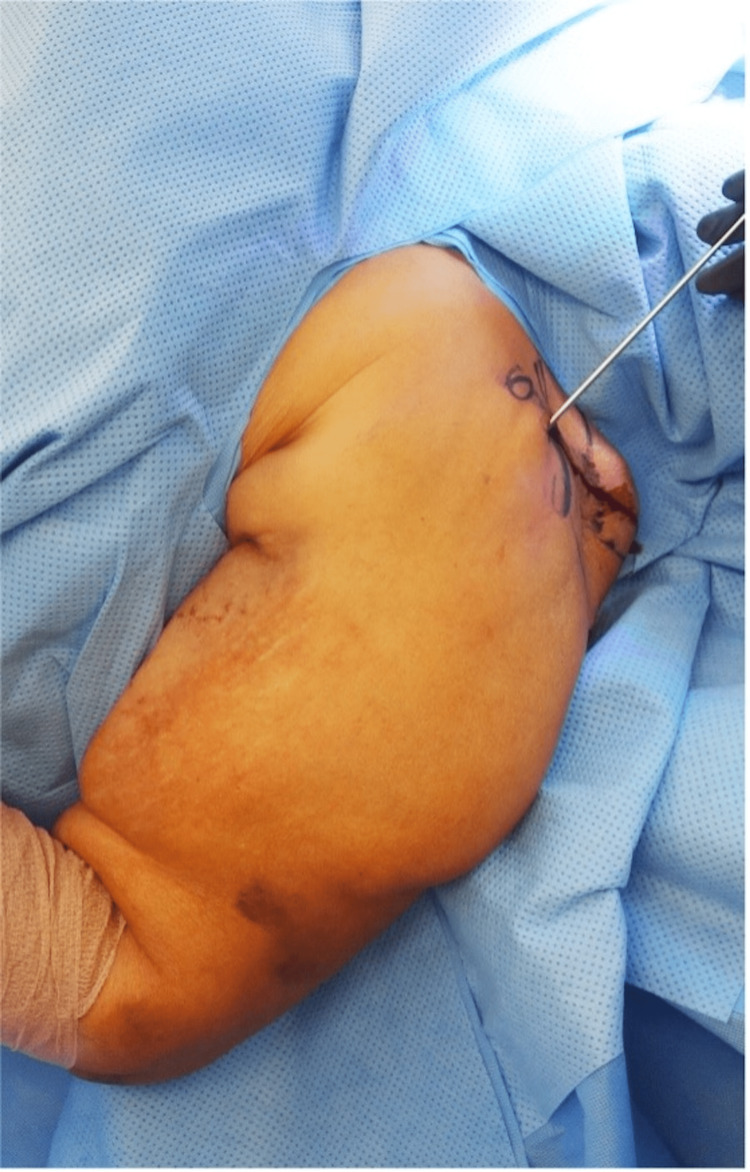
Intraoperative photo showing a shoulder in extension to facilitate guide pin orientation in the sagittal plane.

A guide pin was placed over the skin with the arm in the neutral position so the C-arm could be used to localize the skin penetration site. The shoulder was extended, and a guide pin was percutaneously placed with an entry point medial to the greater tuberosity at the top of the articular surface on the AP view (Figure 3) and centered on the humeral head in the lateral view (Figure 4). Shoulder extension allowed the humeral head to be brought from underneath the acromion, which facilitated center guide pin placement in the lateral view (Figure 5). The medial insertion point was used to avoid violating the tendinous insertion of the rotator cuff [13]. An incision over the guide pin was made just large enough to accommodate the placement of the opening reamer after bluntly spreading through the deltoid. The bony deltoid origin was left undisturbed, and the muscle fibers between the anterior and middle heads were not surgically split.

**Figure 6:**
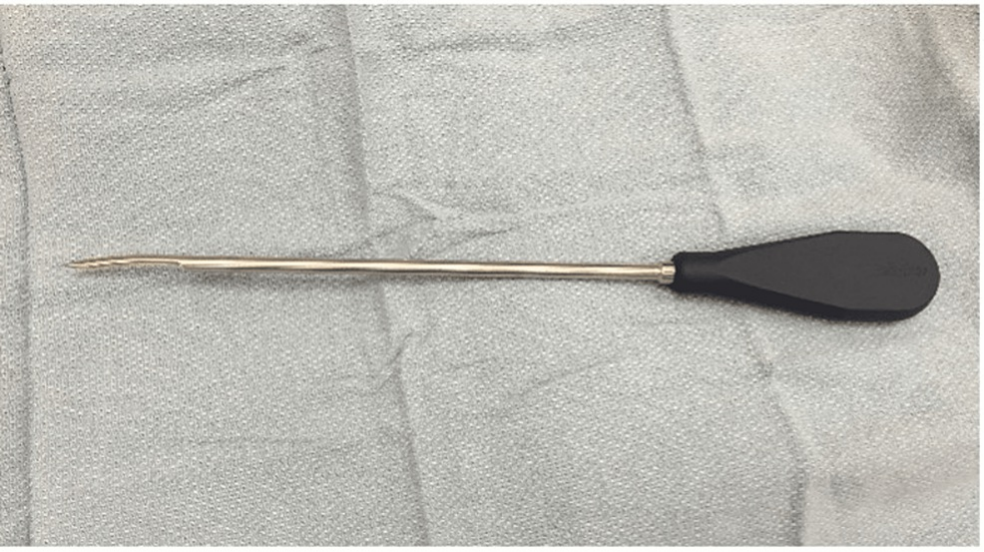
Photograph of an intramedullary rod reduction tool.

**Figure 7:**
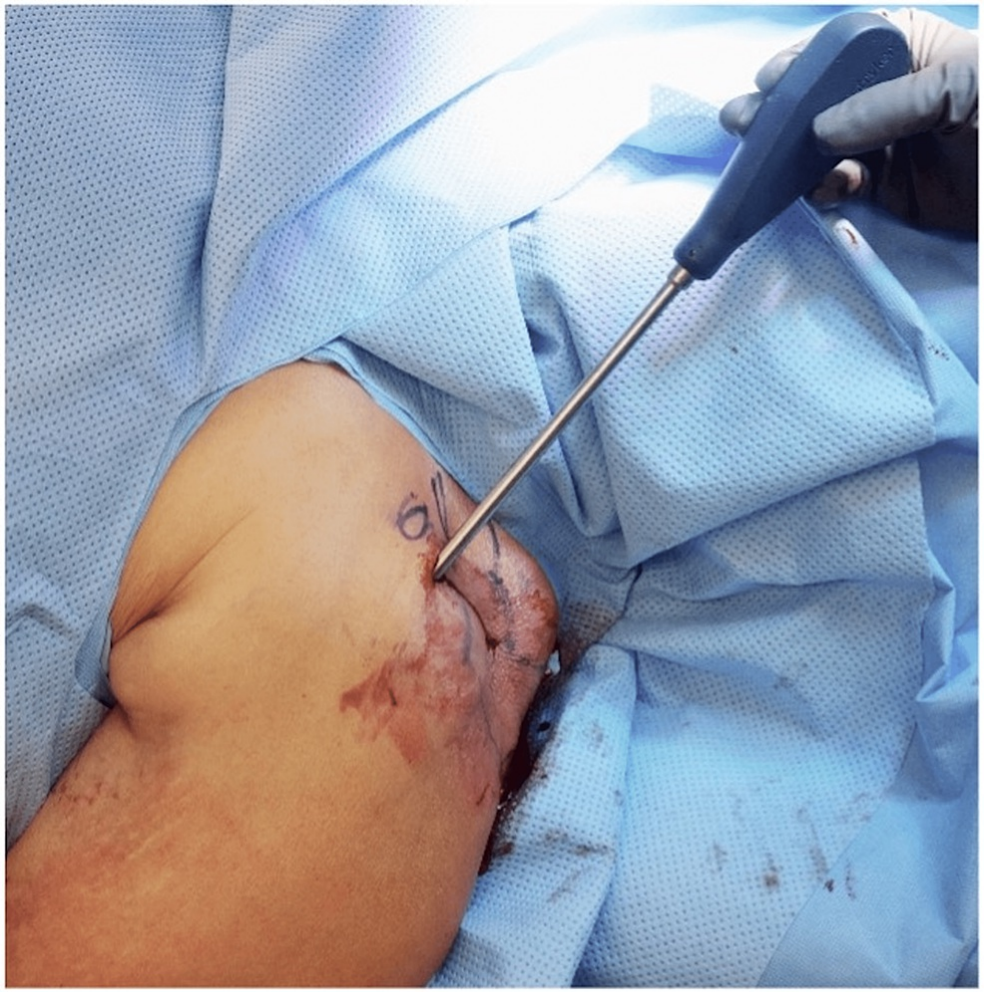
Intraoperative photo revealing placement of the reduction device.

**Figure 8:**
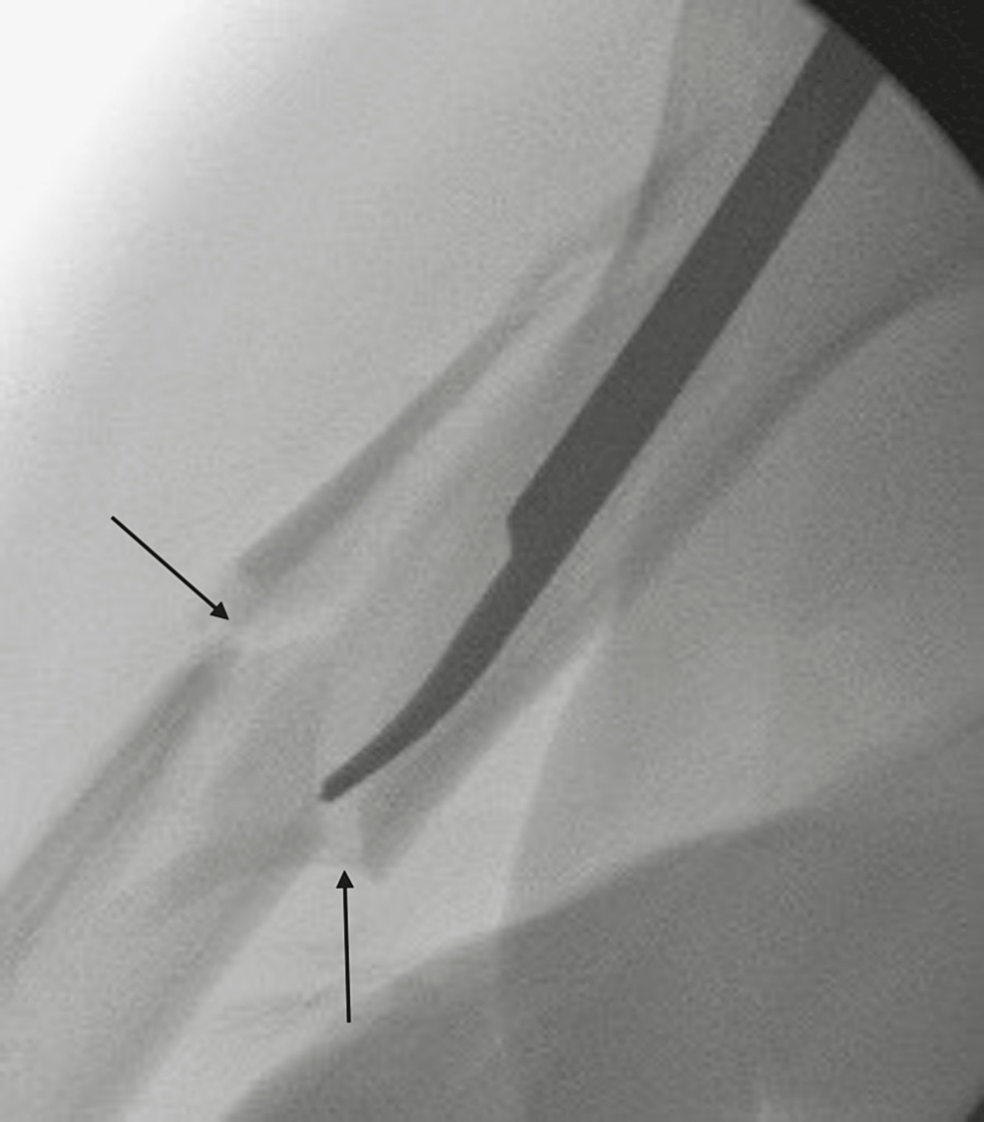
Fluoroscopic image demonstrating the reduction tool aligning the fracture with arrows to depict where the fracture gap is reducing.

**Figure 9:**
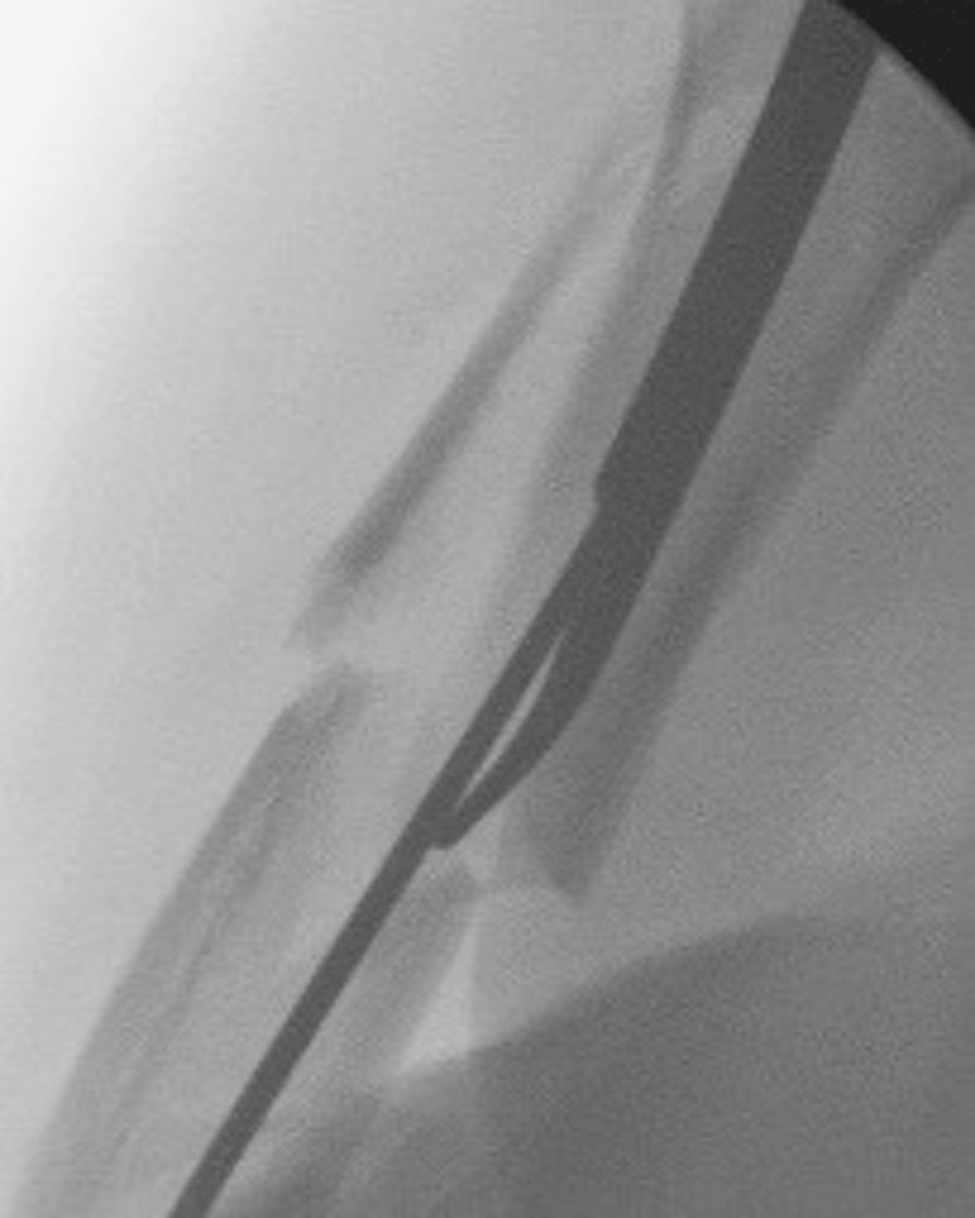
Fluoroscopic image showing the reduction tool to assist in placement of the reaming guide wire.

**Figure 10:**
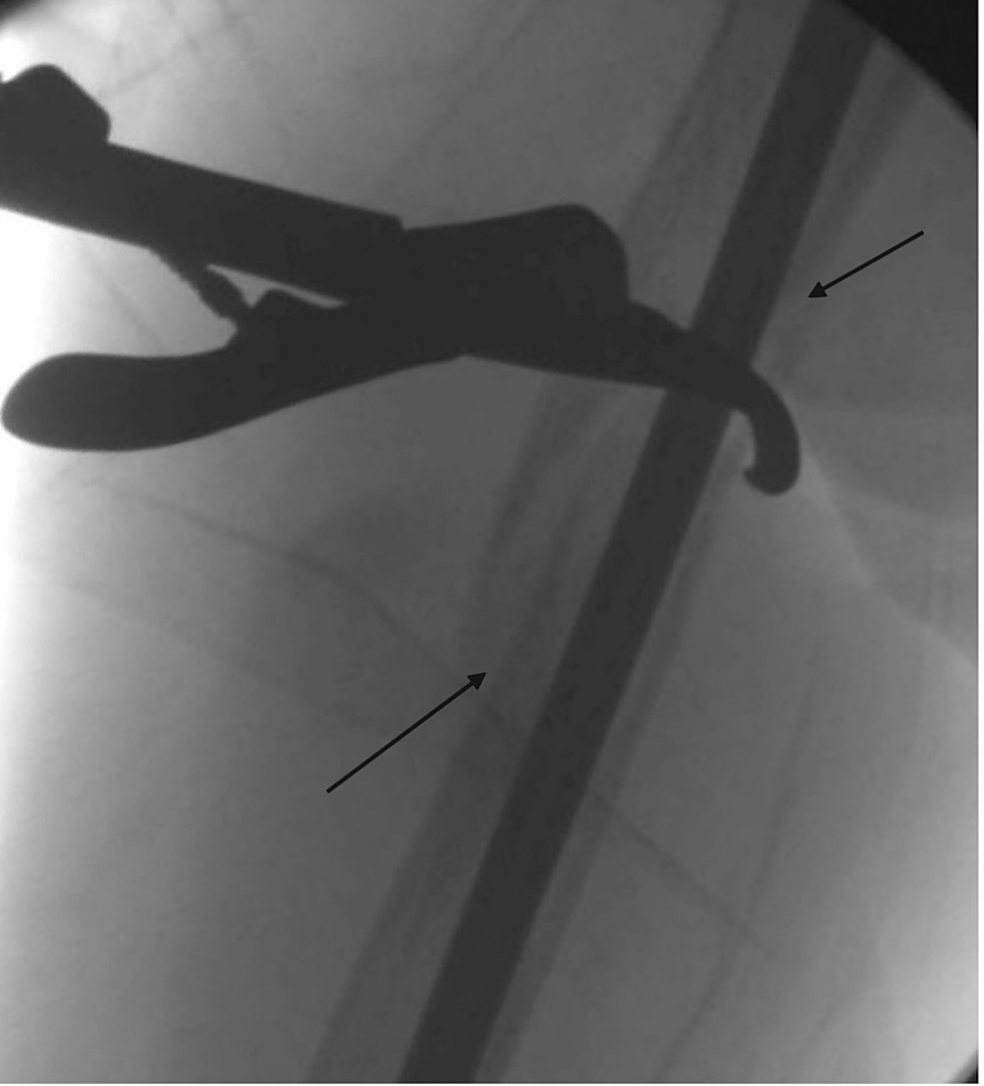
Fluoroscopic image of Lobster clamp being used for percutaneous reduction of proximal diaphyseal fracture. Arrows are used to depict areas where the fracture gap is being reduced.

An intramedullary (IM) reduction rod (Stryker, Kalamazoo, Michigan) was used to facilitate guide wire placement across the fracture sites (Figures 6-7). The combination of gravity and maneuvering of the bone with the IM reduction rod proximally with closed distal manipulation allows for smooth passage of the guide wire across the fracture site (Figures 8-9). When reaming in comminuted cases, power was taken off upon reaching the fracture site, and the reamer was pushed through this area manually, with power being restarted only once in the distal bone fragment to reduce the likelihood of iatrogenic radial nerve injuries. In two cases, percutaneously placed reduction clamps were needed to maintain bone alignment while reaming (Figure 10). This adjunct technique was used in proximal diaphyseal fractures as the deltoid pulled the proximal fragment into excessive abduction, akin to the deformation caused by the gluteus medius and other abductors in subtrochanteric femur fractures [14]. This area, proximal to the deltoid insertion, is safely above where the radial nerve crosses the spiral groove [15]; thus, direct visualization of the nerve was not required. Proximal locking screws were placed using a targeting jig, with the distal locking screws placed freehand from anterior to posterior, with the drill oscillating to prevent slippage and avoid the radial nerve.

## Results

Of the 14 patients in this series, 12 had a clinical and radiographic evaluation at a minimum one-year follow-up, with one patient unable to complete the clinical outcome forms due to a language barrier. Specific fracture pattern analysis using the 2018 AO/OTA Fracture Classification [12] included 2 B2, 1 B3, 5 C2, and 6 C3. There were 12 closed fractures, 1 open fracture due to gunshot wounds, and 1 Gustilo-Anderson grade 3A [16] fracture due to blunt trauma. The mean age of the patient population was 54 (range 37-88) years. Fifty percent (7) of the patients were male, and 50% (7) were polytrauma patients. The average body mass index (BMI) was 34.95. Nine (64.3%) patients were obese (BMI >= 30), seven (50%) had a BMI ≥ 35, and 3 (21.4%) were morbidly obese (BMI 40 and above) (Table 1). Eight of the 14 (57.1%) patients had an Anesthesia Society of America Classification (ASA) Score > 2, and humeral shaft nails were placed as an isolated procedure in 12 of the 14 (85.7%) patients. Of these 12 cases, the average operative time was 103 minutes.

**Table 1:** Demographics: Fracture pattern according to AO Foundation/Orthopaedic Trauma Association (AO/OTA) fracture classification system, gender, polytrauma status, Anesthesia Society of America Classification (ASA) Score > 2, patient age in years (age), body mass index.

	Percent	Fraction
B2 fracture	14.3	(2/14)
B3 fracture	7.1	(1/14)
C2 fracture	35.7	(5/14)
C3 fracture	42.9	(6/14)
Male	50	(7/14)
Polytrauma	50	(7/14)
ASA > 2	57.1	(8/14)
	Average	Std. Dev.
Age	54.07	16.64
BMI	34.95	9.34

**Figure 11:**
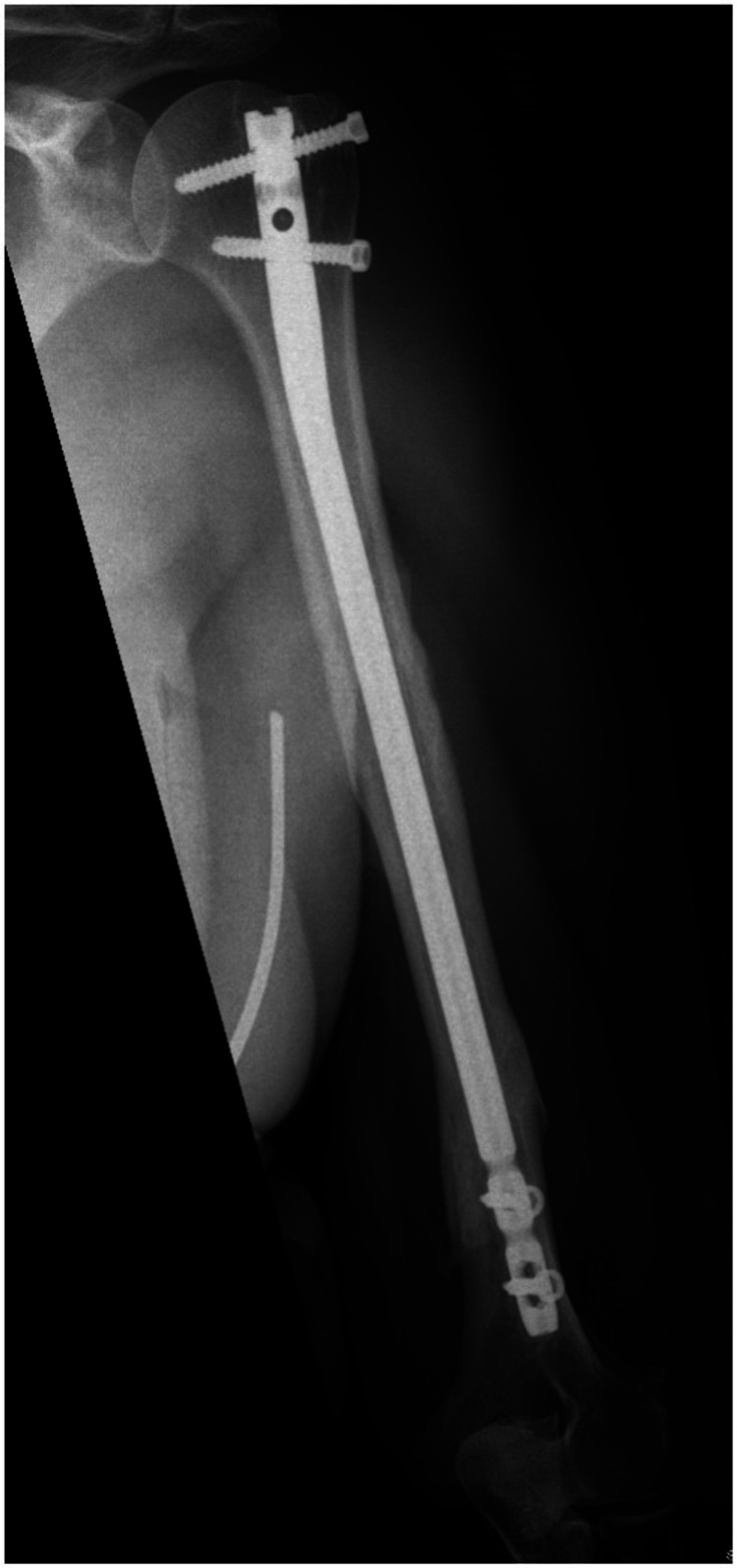
Anterior/posterior humerus radiograph demonstrating a healed segmental fracture at two year follow up.

**Figure 12:**
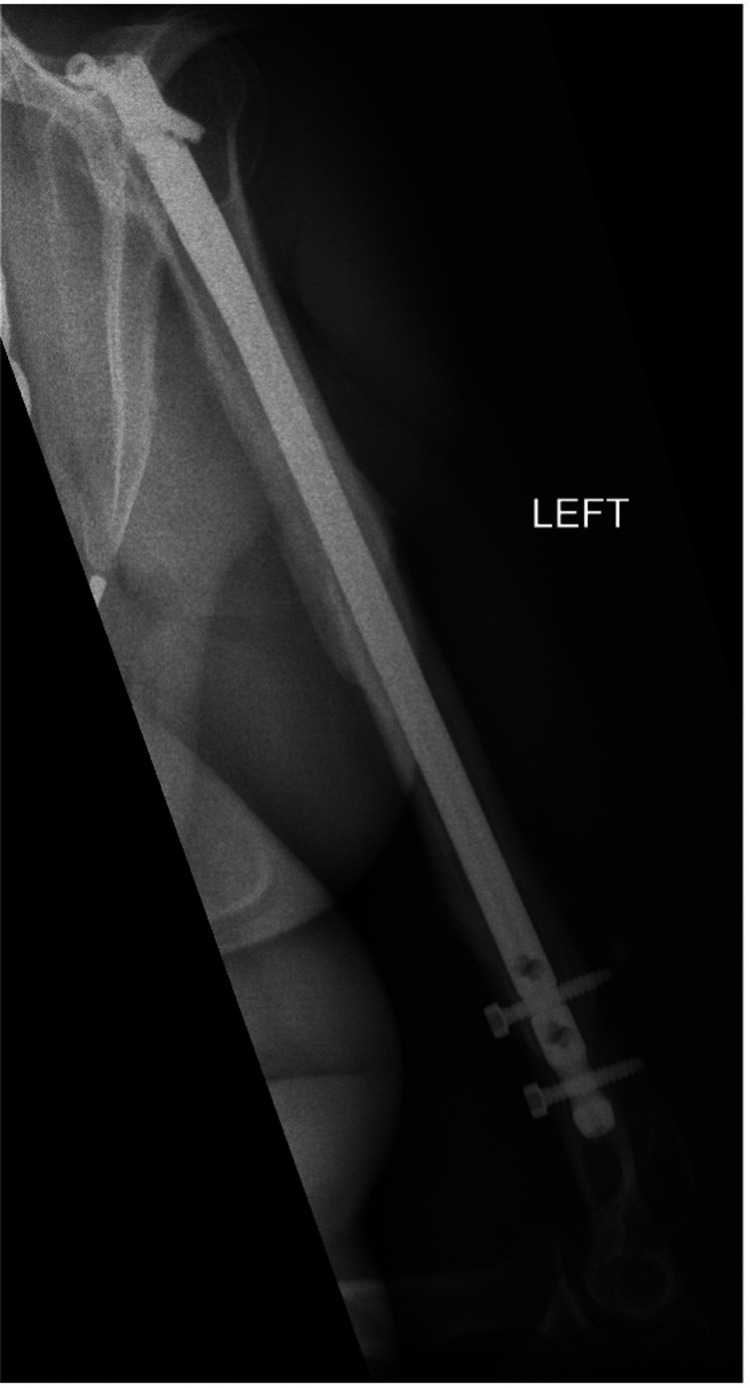
Lateral humerus radiograph demonstrating a healed segmental fracture at two year follow up.

**Figure 13:**
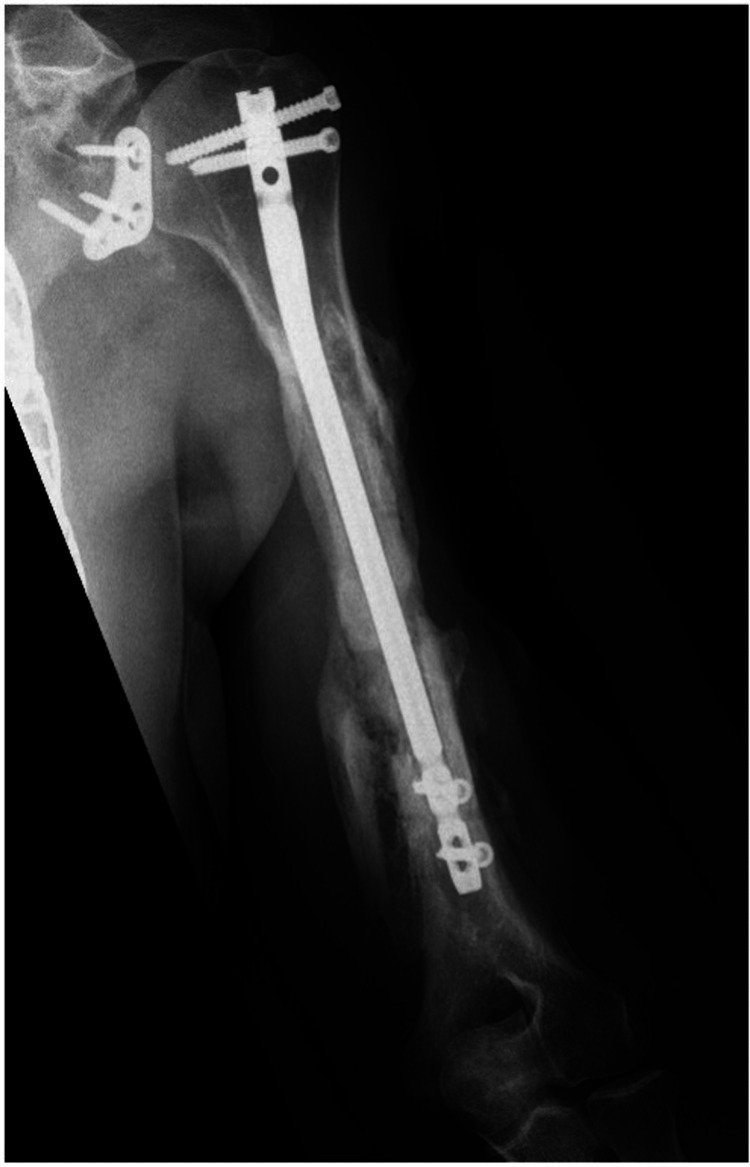
Anterior/posterior humerus radiograph demonstrating a healed comminuted fracture at one year follow up.

**Figure 14:**
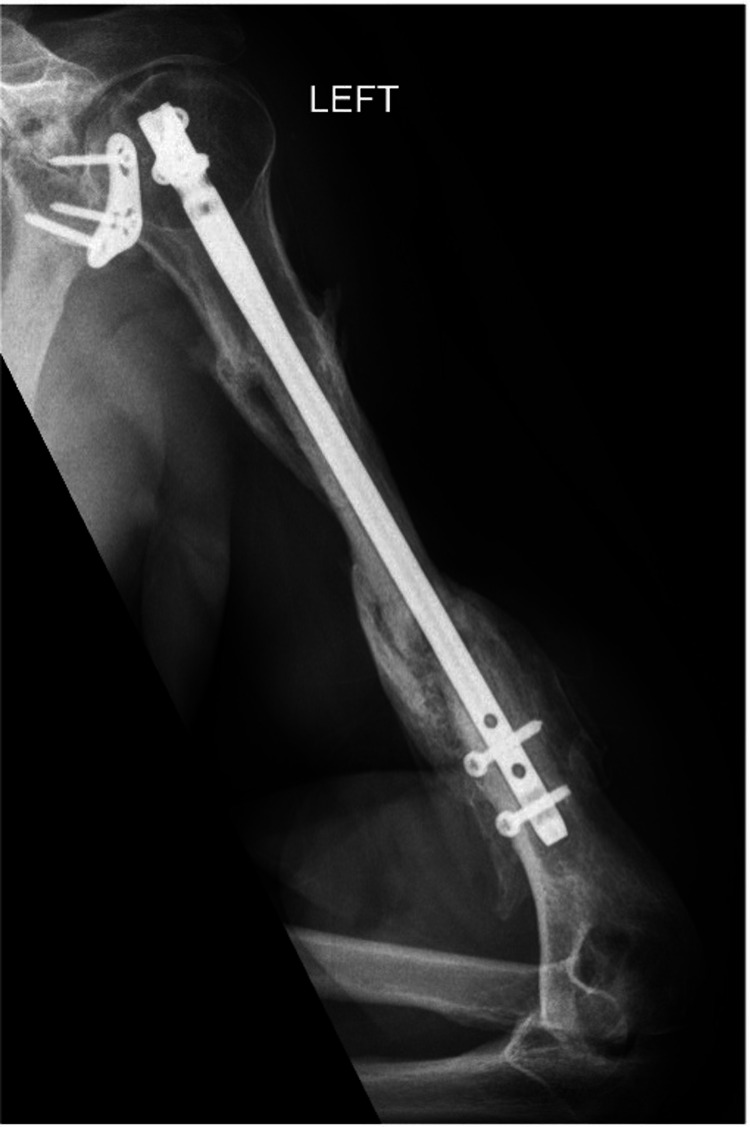
Lateral humerus radiograph demonstrating a healed comminuted fracture at one year follow up.

Fracture union was found in 93% (13/14) of the cases after index percutaneous intramedullary nail placement (Figures 11-14). One patient who had an extremely comminuted fracture with bone loss due to a gunshot wound achieved radiographic union after a secondary plating operation 14 months after the initial nailing procedure, bringing the final fracture union rate to 100%. There were no other secondary operations. There were no preoperative or secondary radial nerve palsies.

Twelve patients had a clinical and radiographic evaluation with a minimum one-year follow-up. One patient was contacted by phone one year postoperatively and reported using the arm for daily activities and has not undergone any secondary operations. The final patient was a migrant who was lost to follow-up after the radiographic union.

Average clinical outcome scores collected at a minimum of one-year follow-up were American Shoulder and Elbow Society (ASES) 78.2, Constant 72.1, Single Assessment Numeric Evaluation (SANE) 81.9, and Penn Shoulder (PENN) 82.7 (Table 2).

**Table 2:** Outcomes: primary union rate, secondary union rate, radial nerve palsy, operative time in minutes (time), American Shoulder and Elbow Society (ASES), Constant, Penn shoulder (PENN), and Single Assessment Numeric Evaluation (SANE) at minimum one year after surgery.

	Percent	Fraction
Primary union	92.9	(13/14)
Secondary union	100.0	(14/14)
Radial nerve palsy	0	(0/14)
	Average	Std. Dev.
Operative time	102.75	21.02
ASES	78.15	12.75
Constant	72.08	13.48
PENN	81.87	12.21
SANE	82.73	13.71

## Discussion

This is a clinical and radiographic report of consecutive complex humeral diaphyseal fractures treated with percutaneous IMN placement with nearly 80% AO/OTA type C fractures in a high-risk patient population. Radiographic union was achieved after the index procedure in all but one patient. The clinical outcome scores in this series demonstrate satisfactory shoulder function at a one-year follow-up.

The union rate after the index procedure in this series was 93% (13/14) and compares favorably to union rates previously reported in the literature (77-95%) with plate and nail fixation of humeral shaft fractures [10,11,17,18]. In addition, while previous reports have included a wide array of fracture patterns, this study focused primarily on complex fracture patterns. The significance of increasingly comminuted fracture patterns is that they often rely on secondary bone healing to achieve union. IMNs are ideal in this scenario as they allow the surgeon to control length, alignment, and rotation with minimal detriment to the soft tissue envelope. Percutaneous insertion further minimizes soft tissue disruption, allowing healing potential to be maximized.

Compared to open plating, the main advantages of IMN pertain to its decreased need for dissection, which leads to decreased operative time [9,19], decreased soft tissue disruption [2,5], and decreased potential for radial nerve palsy [6,7]. Fan et al. [19] found blood loss, operative time, and hospital stay to be significantly lower for patients who underwent intramedullary nail fixation when compared to locked compression plates for humerus fractures. In a prospective randomized study, Chapman et al. [17] concluded both IMN and compression plating provided predictable methods for fracture union, but their opinion was that antegrade nails were advantageous in more comminuted type C fractures.

Although minimally invasive plate osteosynthesis (MIPO) has recently gained popularity in treating humeral shaft fractures [20], it remains unclear how effective MIPO is in treating extensively comminuted fractures. In a randomized trial comparing MIPO and IMN, Benegas et al. [10] found both techniques produced similar union rates and shoulder outcome scores, despite type C fracture patterns being over three times more prevalent in the IMN cohort. In fractures with distal metaphyseal extension, such as those in Figure 1A-1B, it may be difficult to obtain adequate plate fixation in the distal fragment; in contrast, the IMN used in this study allows an interlocking screw to be placed 7.5 mm and 21 mm proximal to the distal end of the implant [21]. From a rehabilitation standpoint, surgeons may be less willing to allow patients to weight bear immediately after plate fixation in these complex fracture patterns, which is significant in polytrauma patients. The reduction of complex fractures may also be difficult in MIPO due to the inability to control intercalary fragments. Finally, although traditional IMN and MIPO may have similar incision sizes, the present described percutaneous IMN technique utilizes much smaller incisions than the 4-5 cm proximal and distal incisions described in MIPO [22,23].

Importantly, there were no cases of postoperative radial nerve palsy using the described technique. Similarly, Fan et al. [19] reported no radial nerve palsies in the IMN group compared to 10% in the open-plating group. Other studies looking at secondary radial nerve palsies in humeral shaft fractures have found a majority to occur in patients who underwent open plate fixation [8,24]. The nerve retraction typically needed for lateral plate placement is avoided with IMN placement. Radial nerve exploration is always an option during humeral nailing. However, the advantages of a minimally invasive nailing technique are partially negated by the exposure needed for nerve exploration.

The percutaneous technique in this study differs from previously described methods of intramedullary nailing placement for humeral shaft fractures, which involve splitting of the deltoid muscle or detaching it from its origin and a longitudinal incision of the rotator cuff [10,11,17], all of which can lead to longer surgeries and more blood loss, especially in patients with larger body habitus. The percutaneous stab incisions decrease the area susceptible to surgical site infection, a complication that is also often increased in obese patients [25]. The present percutaneous technique does not involve surgical cutting of the deltoid and supraspinatus but rather a blunt spreading of the deltoid and a single percutaneously placed "poke hole" through the muscular portion of the rotator cuff to reach the insertion point on the humeral head. The nail is inserted into a non-articulating portion of the chondral surface of the humeral head, previously described as the optimal starting point to avoid rotator cuff disruption [13,26]. This technique allows the muscular portion of the supraspinatus to be penetrated while sparing the tendinous insertion, as is done in arthroscopic labral repairs [27]. While conceptual concerns exist regarding penetrating the articular cartilage of the humeral head, reports documenting the use of retrograde femoral nails with a starting point through the non-weight-bearing articular cartilage of the distal femur date back over 20 years [28,29] and continue to yield positive results [30].

There are several limitations to this study. First, this cohort is retrospective and does not have a comparison group with which to contrast endpoints. Additionally, the relatively small sample size (n=14) renders it underpowered. Finally, the follow-up time of one year may not capture later long-term sequelae. However, this is a unique cohort of complex fracture patterns in higher-risk obese and polytrauma patients with radiographic and clinical outcomes showing a high union rate and low complications using a previously undescribed percutaneous surgical technique.

## Conclusions

In a consecutive series of complex fracture patterns in patients, percutaneous intramedullary nailing of complex humeral diaphyseal fractures produced high union rates and satisfactory clinical outcome scores, even in obese and polytrauma patients. Percutaneous placement of IMN should be considered as a treatment option in multifragmentary comminuted, segmental, and long wedge humeral shaft fractures.

## References

[1] Tsai CH, Fong YC, Chen YH, Hsu CJ, Chang CH, Hsu HC (2009). The epidemiology of traumatic humeral shaft fractures in Taiwan. Int Orthop.

[2] Ouyang H, Xiong J, Xiang P, Cui Z, Chen L, Yu B (2013). Plate versus intramedullary nail fixation in the treatment of humeral shaft fractures: an updated meta-analysis. J Shoulder Elbow Surg.

[3] Pollock FH, Drake D, Bovill EG (1981). Treatment of radial neuropathy associated with fractures of the humerus. J Bone Joint Surg Am.

[4] Foster RJ, Swiontkowski MF, Bach AW, Sack JT (1993). Radial nerve palsy caused by open humeral shaft fractures. J Hand Surg.

[5] Gregory PR, Sanders RW (1997). Compression plating versus intramedullary fixation of humeral shaft fractures intramedullary fixation of humeral shaft fractures. J Am Acad Orthop Surg.

[6] Heineman DJ, Bhandari M, Poolman RW (2012). Plate fixation or intramedullary fixation of humeral shaft fractures--an update. Acta Orthop.

[7] Flinkkilä T, Hyvönen P, Siira P, Hämäläinen M (2004). Recovery of shoulder joint function after humeral shaft fracture: a comparative study between antegrade intramedullary nailing and plate fixation. Arch Orthop Trauma Surg.

[8] Zhao JG, Wang J, Meng XH, Zeng XT, Kan SL (2017). Surgical interventions to treat humerus shaft fractures: a network meta-analysis of randomized controlled trials. PLoS One.

[9] Chen F, Wang Z, Bhattacharyya T (2013). Outcomes of nails versus plates for humeral shaft fractures: a Medicare cohort study. J Orthop Trauma.

[10] Benegas E, Ferreira Neto AA, Gracitelli ME (2014). Shoulder function after surgical treatment of displaced fractures of the humeral shaft: a randomized trial comparing antegrade intramedullary nailing with minimally invasive plate osteosynthesis. J Shoulder Elbow Surg.

[11] Patino JM (2015). Treatment of humeral shaft fractures using antegrade nailing: functional outcome in the shoulder. J Shoulder Elbow Surg.

[12] Meinberg EG, Agel J, Roberts CS, Karam MD, Kellam JF (2018). Fracture and dislocation classification compendium-2018. J Orthop Trauma.

[13] Boileau P, d'Ollonne T, Bessière C, Wilson A, Clavert P, Hatzidakis AM, Chelli M (2019). Displaced humeral surgical neck fractures: classification and results of third-generation percutaneous intramedullary nailing. J Shoulder Elbow Surg.

[14] Yoon RS, Donegan DJ, Liporace FA (2015). Reducing subtrochanteric femur fractures: tips and tricks, do's and don'ts. J Orthop Trauma.

[15] Carlan D, Pratt J, Patterson JM, Weiland AJ, Boyer MI, Gelberman RH (2007). The radial nerve in the brachium: an anatomic study in human cadavers. J Hand Surg Am.

[16] Gustilo RB, Anderson JT (1976). Prevention of infection in the treatment of one thousand and twenty-five open fractures of long bones: retrospective and prospective analyses. J Bone Joint Surg Am.

[17] Chapman JR, Henley MB, Agel J, Benca PJ (2000). Randomized prospective study of humeral shaft fracture fixation: intramedullary nails versus plates. J Orthop Trauma.

[18] Kulkarni VS, Kulkarni MS, Kulkarni GS, Goyal V, Kulkarni MG (2017). Comparison between antegrade intramedullary nailing (IMN), open reduction plate osteosynthesis (ORPO) and minimally invasive plate osteosynthesis (MIPO) in treatment of humerus diaphyseal fractures. Injury.

[19] Fan Y, Li YW, Zhang HB (2015). Management of humeral shaft fractures with intramedullary interlocking nail versus locking compression plate. Orthopedics.

[20] Davies G, Yeo G, Meta M, Miller D, Hohmann E, Tetsworth K (2016). Case-match controlled comparison of minimally invasive plate osteosynthesis and intramedullary nailing for the stabilization of humeral shaft fractures. J Orthop Trauma.

[21] Stryker Stryker (2023). T2 humeral nailing system technique guide. 2017. Stryker.

[22] Apivatthakakul T, Arpornchayanon O, Bavornratanavech S (2005). Minimally invasive plate osteosynthesis (MIPO) of the humeral shaft fracture. Is it possible? A cadaveric study and preliminary report. Injury.

[23] Apivatthakakul T, Phornphutkul C, Laohapoonrungsee A, Sirirungruangsarn Y (2009). Less invasive plate osteosynthesis in humeral shaft fractures. Oper Orthop Traumatol.

[24] Wang JP, Shen WJ, Chen WM, Huang CK, Shen YS, Chen TH (2009). Iatrogenic radial nerve palsy after operative management of humeral shaft fractures. J Trauma.

[25] Yuan K, Chen HL (2013). Obesity and surgical site infections risk in orthopedics: a meta-analysis. Int J Surg.

[26] Hatzidakis AM, Shevlin MJ, Fenton DL, Curran-Everett D, Nowinski RJ, Fehringer EV (2011). Angular-stable locked intramedullary nailing of two-part surgical neck fractures of the proximal part of the humerus. A multicenter retrospective observational study. J Bone Joint Surg Am.

[27] Myer DM, Caldwell PE 3rd (2012). ORV arthroscopic transosseous bony Bankart repair. Arthrosc Tech.

[28] Moed BR, Watson JT (1999). Retrograde nailing of the femoral shaft. J Am Acad Orthop Surg.

[29] Sanders R, Koval KJ, DiPasquale T, Helfet DL, Frankle M (1993). Retrograde reamed femoral nailing. J Orthop Trauma.

[30] Daglar B, Gungor E, Delialioglu OM (2009). Comparison of knee function after antegrade and retrograde intramedullary nailing for diaphyseal femoral fractures: results of isokinetic evaluation. J Orthop Trauma.

